# An Unusual Hodgkin's Lymphoma Journey: Cardiac Tamponade, Primary Refractoriness, Immune Thrombocytopenia, and Post-Traumatic Stress Disorder

**DOI:** 10.7759/cureus.15341

**Published:** 2021-05-30

**Authors:** Juan Jose Chango Azanza, James Vredenburgh

**Affiliations:** 1 Internal Medicine, University of Connecticut School of Medicine, Farmington, USA; 2 Hematology and Oncology, St. Francis Hospital and Medical Center, Hartford, USA

**Keywords:** hodgkin lymphoma, cardiac tamponade, immune thrombocytopenia, post-traumatic stress disorder

## Abstract

Hodgkin’s lymphoma (HL) is a lymphoid neoplasm in which malignant Hodgkin or Reed-Sternberg cells are present in tissues mixed with heterogeneous non-malignant inflammatory cells. Pericardial effusion (PEEF) in HL is rare. Furthermore, hemodynamically significant effusions causing cardiac tamponade (CTp) are exceedingly uncommon. CTp as the initial presentation of HL is extremely rare. We describe the case of a 21-year-old man who presented with CTp requiring pericardiocentesis. On further workup, he was found to have a large mediastinal mass with a biopsy consistent with classic HL. His clinical course was complicated by the development of immune thrombocytopenia (ITP) and post-traumatic stress disorder (PTSD).

## Introduction

Cardiac involvement in Hodgkin’s lymphoma (HL) can occur in two forms: primary cardiac lymphomas and secondary or disseminated lymphomas. Pericardial effusions (PEEF) occur in less than 5% of primary lymphomas [1]. However, PEEFs are diagnosed in up to 20% of disseminated lymphomas [2]. Cardiac tamponade (CTp) is exceedingly rare and only a few cases have been described with both HL and non-HL. We present a patient who developed CTp as the initial presentation of classic HL. His clinical course was complicated by immune thrombocytopenia (ITP) and post-traumatic stress disorder (PTSD).

## Case presentation

A 21-year-old man with a history of hypertension presented to the emergency room with a four-day history of worsening exertional dyspnea, chest pressure, and orthopnea. He had experienced a non-productive cough and exertional shortness of breath for one month. He denied fever, night sweats, chills, decreased appetite, weight loss, sore throat, rhinorrhea, abdominal pain, abdominal distention, diarrhea, hematuria, hematochezia, or melena. He denied recent infections or travel. His physical examination was relevant for neck fullness on inspection with dilated neck veins and varicosities. Bilateral cervical and axillary adenopathies were felt on palpation. There was dullness to percussion at the bilateral lung bases with clear lungs to auscultation. Heart sounds were muffled. His laboratory workup showed a white blood cell count of 11.4 K/µL (normal value 3.8-10.6 × K/µL), absolute neutrophil count of 8.8 K/µL (1.8-7.8 K/µL), hemoglobin 11.4 GM/DL (13.5-18 GM/DL), platelets 467 K/µL (150-450 K/µL). Chemistries were unremarkable. 

**Figure 1:**
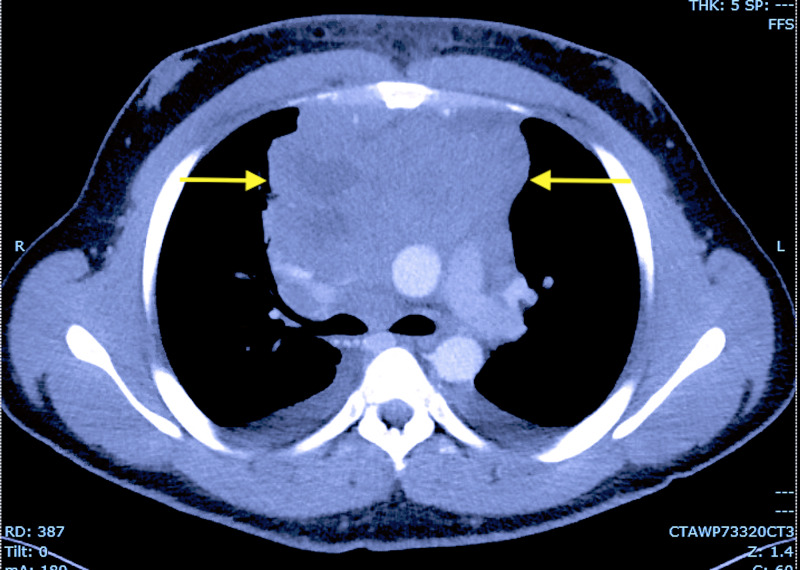
Computed tomography of the chest showing a large anterior mediastinal mass (yellow arrows) with compression of the superior vena cava.

**Figure 2:**
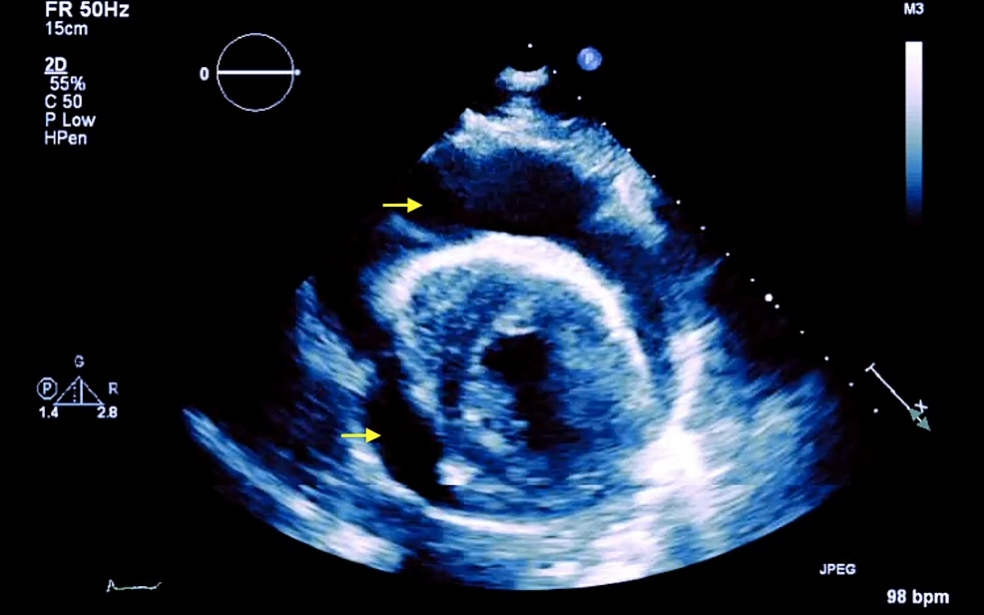
Transthoracic echocardiogram showing a large pericardial effusion (yellow arrows) in the setting of cardiac tamponade.

A computed tomography (CT) of the chest showed a large superior mediastinal mass causing superior vena cava compression (Figure 1). There were multiple necrotic lymphadenopathies in the mediastinum, pre-tracheal space, subcarinal region, hila, and axillae. Thickening of the pericardium consistent with a PEEF was appreciated. An echocardiogram done showed a hemodynamically significant large PEEF with collapse of the right atrium and ventricle consistent with CTp (Figure 2).

The PEEF was treated with pericardiocentesis and a pericardial drain. Fluid analysis was negative for infection and malignancy. His pericardial drain was left in place for a few days and he did not develop recurrence of the effusion. Therefore, his drain was removed. He underwent a CT-guided biopsy of the mediastinal mass which was consistent with nodular sclerosing HL stage IIb. He was treated initially with six cycles of ABVD chemotherapy (doxorubicin, bleomycin, vinblastine, and dacarbazine). Unfortunately, his positron emission tomography (PET) scan showed early disease progression after cycle six of ABVD. The patient became frustrated and overwhelmed by his treatment. He was quite disturbed by the negative chemotherapy response. He received two cycles of ICE chemotherapy (ifosfamide, carboplatin, and etoposide) and two cycles of GVD (gemcitabine, vinorelbine, and doxorubicin). He achieved a near-complete response with GVD and received an allogeneic transplant. Consolidation radiotherapy was provided to increase his probability of cure.

Unfortunately, six months later, the patient developed shortness of breath and wheezing. A repeat PET scan showed recurrence of disease which was confirmed by a biopsy. He was treated with brentuximab vedotin with a good initial response. Nonetheless, recurrence was noted again in a repeat PET scan with new right upper and middle lung masses, right loculated pleural effusion, and multiple lymphadenopathies. At that time, nivolumab (a programmed-death ligand 1 or PDL-1 inhibitor) was initiated showing excellent clinical and radiographic response and complete resolution of his disease. Additionally, he developed ITP (secondary causes of thrombocytopenia were ruled out) effectively treated with intravenous immunoglobulin and romiplostim.

Since his diagnosis of HL, the patient developed anxiety, intrusive memories, nightmares about his cancer diagnosis, flashbacks, and other symptoms consistent with PTSD. The development of any symptom such as a headache or abdominal pain distressed him profoundly requesting workup for an undiagnosed tumor. He developed a fear of disease recurrence. Sadly, the severity of his symptoms affected his daily activities. His PTSD was managed with a multidisciplinary approach including behavioral health, psychiatry, social work, and other providers. He was offered psychotherapy and pharmacotherapy. Fortunately, he has remained free of disease for more than four years.

## Discussion

HL is a rare B-cell malignancy that represents approximately 11% of all lymphomas in the United States [3]. HL is divided into classic HL and nodular lymphocyte-predominant HL. Classic HL is subdivided into different types such as nodular sclerosis, mixed cellularity, lymphocyte depletion, and lymphocyte-rich Hodgkin lymphoma [3]. HL has a bimodal age distribution, with an increased incidence in the second and fifth decades of life [4]. PEEFs occur in less than 5% of primary lymphomas [1]. However, PEEFs and cardiac metastasis are diagnosed in up to 20% of disseminated lymphomas [2]. Cardiac involvement in malignancy can occur via the hematogenous, lymphatic, venous, and contiguous spread. The pericardium is the most frequently affected site of cardiac metastasis, particularly due to lymphomas [5]. The clinical manifestations of cardiac involvement in lymphomas are multiple. Patients may remain asymptomatic or develop heart failure (shortness of breath, orthopnea), arrhythmia, pericarditis (chest pain). 

Diagnosis of cardiac involvement in lymphomas can be achieved by indirect (imaging modalities) and direct (pathology) techniques. There is a role of imaging in the diagnosis of cardiac involvement by transthoracic echocardiography (TTE) and magnetic resonance imaging (MRI) which can identify pericardial lesions and borders with a better definition [6]. The pathologic diagnosis can be achieved by pericardial fluid analysis and/or pericardial/epicardial biopsy [2]. In our case, the initial pericardial fluid analysis did not show signs of lymphoma or infection. However, given the newly found mass consistent with HL with no other clear cause of his effusion, it was believed to be related to his HL. His PEEF was recurrent which was also an important feature to correlate with his malignancy. Our patient developed a primary refractory disease which is defined as progression or no response during induction treatment or within 90 days of completing therapy. In these cases, the clinical course is grim. Second-line chemotherapy generally has low response rates and long-term disease-free survival of 5%-10% [7].

Treatment of CTp in HL includes pericardiocentesis, systemic antineoplastic therapy, and consideration of intrapericardial instillation of cytostatic/sclerotic agents to prevent a recurrence. CTp is a class I indication for pericardiocentesis [6]. Radiation therapy may have good results in lymphomas but increases the risk for pericarditis and myocarditis [8]. If pericardiocentesis cannot be performed, pericardiotomy can be considered but has a higher risk of complications. A pericardial window is safe and effective in the management of malignant CTp [8]. Patients with HL stage IIb or higher (advanced-stage HL) are usually treated with combination chemotherapy alone [3]. ABVD remains the most widely used treatment in the United States for advanced-stage HL [7]. The escalated bleomycin, etoposide, doxorubicin, cyclophosphamide, vincristine, procarbazine, and prednisolone (eBEACOPP) regimen devised by the German Hodgkin study group (GHSG) can also be considered but is not recommended in patients >60 years old as side effect profile is higher than ABVD [9]. HL may be refractory or relapsing in about 20%-30% of patients [10]. Favored salvage regimens include ifosfamide, carboplatin, and etoposide (ICE), epirubicin in place of carboplatin (IVE), and cytarabine and cisplatin (DHAP). If a complete metabolic response is achieved, the next step would be autologous stem cell transplantation.

Our patient developed a refractory and relapsed disease. Therapeutic options for those situations include targeting the cluster of differentiation 30 (CD30) molecule if expressed in the Reed-Sternberg cells of HL with agents such as brentuximab vedotin [11]. Immune checkpoint inhibitors are important medications in the treatment of refractory and relapsed HL. Blockade of the PDL-1 molecule has demonstrated remarkable objective response rates of 65% to 87% and durable disease control in phase 1 and 2 trials [12]. The two most studied PDL-1 inhibitors in the treatment of HL are pembrolizumab and nivolumab. Fortunately, our patient responded well to nivolumab after a challenging therapeutic journey. Interestingly, the prevalence of PTSD in a retrospective study was not significantly higher in HL survivors (13%) when compared to sibling controls (6.9%, p = .098. However, a significantly larger proportion of survivors (35.2%) met the criteria for partial-PTSD compared to siblings (17.8%, p = .004) [13]. Patients with HL and partial-PTSD and PTSD can have a higher functional impairment [14].

## Conclusions

CTp can be the first presenting feature of classic HL. These cases can be challenging to diagnose and treat. Systemic chemotherapy has low response rates and long-term disease-free survival. As described in our case, management can be challenging requiring multiple therapeutic regimens. Complications are multiple and related to malignancy and its treatment. After multiple regimens, our patient responded well to PDL-1 inhibition with nivolumab. He developed primary refractory and relapsing disease with complications including ITP and PTSD. His PTSD has become the main health issue in his life after his long disease course. This case demonstrates the importance of considering malignancy in patients presenting with CTp or PEEF. Furthermore, we can anticipate more difficulties with treatment and complications. Understanding the psychological aspects of the disease is important in monitoring these patients.
